# Phylogeny, Pathogenicity, Transmission, and Host Immune Responses of Four H5N6 Avian Influenza Viruses in Chickens and Mice

**DOI:** 10.3390/v11111048

**Published:** 2019-11-10

**Authors:** Yafen Song, Weiqiang Li, Wenbo Wu, Zhiting Liu, Zhuoliang He, Zuxian Chen, Bingbing Zhao, Siyu Wu, Chenghuai Yang, Xiaoyun Qu, Ming Liao, Peirong Jiao

**Affiliations:** 1 College of Veterinary Medicine, South China Agricultural University, Guangzhou 510642, China; songyafen1@hotmail.com (Y.S.); wqli0815@gmail.com (W.L.); wenbo0816@foxmail.com (W.W.); liuzhiting1995@foxmail.com (Z.L.); hezhuoliang@foxmail.com (Z.H.); czx0216@outlook.com (Z.C.); zbingbing2016@outlook.com (B.Z.); 2016102809@stu.scau.edu.cn (S.W.); qxy0926@126.com (X.Q.); 2 China Institute of Veterinary Drug Control, Beijing 100081, China; ychenghuai@163.com

**Keywords:** H5N6 avian influenza virus, evolution, pathogenicity, transmission, immune responses, chicken, mice

## Abstract

H5Nx viruses have continuously emerged in the world, causing poultry industry losses and posing a potential public health risk. Here, we studied the phylogeny, pathogenicity, transmission, and immune response of four H5N6 avian influenza viruses in chickens and mice, which were isolated from waterfowl between 2013 and 2014. Their HA genes belong to Clade 2.3.4.4, circulated in China since 2008. Their NA genes fall into N6-like/Eurasian sublineage. Their internal genes originated from different H5N1 viruses. The results suggested that the four H5N6 viruses were reassortants of the H5N1 and H6N6 viruses. They cause lethal infection with high transmission capability in chickens. They also cause mild to severe pathogenicity in mice and can spread to the brain through the blood–brain barrier. During the infection, the viruses result in the up-regulation of PRRs and cytokine in brains and lungs of chickens and mice. Our results suggested that the high viral loads of several organs may result in disease severity in chickens and mice; there were varying levels of cytokines induced by the H5N6 viruses with different pathogenicity in chickens and mice.

## 1. Introduction

Influenza A viruses, possessing eight-segmented negative-sense RNAs as their genome, have been isolated from many species including humans, pigs, horses, mink, felids, dogs, marine mammals, wild birds and domestic birds [1,2]. Based on their ability to cause disease, avian influenza viruses (AIVs) can also be divided into highly pathogenic avian influenza (HPAI) viruses and low pathogenic avian influenza (LPAI) viruses [1,3]. H5N1 HPAI viruses have caused numerous outbreaks in poultry in over 60 countries of Asia, Europe, and Africa [4]. From 25 November 2003 to 27 September 2019, 861 confirmed human cases of avian influenza A (H5N1) virus infection have been reported to WHO from 16 countries including China, Vietnam, Thailand, 455 of which have died [5]. Therefore, H5N1 HPAI viruses still continue to pose a threat to human health and the poultry industry.

Since 2003, H5N1 HPAI viruses of the A/goose/Guangdong/1/96 lineage (Gs/GD-lineage) have become enzootic in some countries and continued to cause outbreaks in poultry and sporadic human infections. The Gs/GD-lineage H5N1 HPAI viruses have undergone extensive evolution giving rise to 10 clades (0–9) with some of those clades containing subclades. Recently, Clade 2.3.4.4 viruses, composed of a variety of H5 viruses with different NA subtypes (N1, N2, N3, N5, N6, and N8), have been detected from human, birds, and environmental samples in 47 countries in Africa, Asia, and Europe [6,7,8]. Human infection with the H5N6 avian influenza virus (AIV) belonging to Clade 2.3.4.4 was first reported in the Sichuan province in May 2014 and subsequently reported in Guangdong, Yunnan, Hubei, Anhui, Hunan, and Guangxi in China [9,10,11,12]. Up to 27 September 2019, a total of 24 laboratory-confirmed cases of human infection with influenza A(H5N6) virus have been reported to WHO [13].

There are many factors contributing to viral pathogenesis of avian influenza virus, including virus and host factors [14]. After AIV infection, the virus may use different strategies to evade the host response and to replicate in host cells. Host responses to the avian influenza virus involve humoral and cell mediated immune responses [15]. Many studies have demonstrated that the innate immune response plays an important role in the pathogenesis of H5N1 influenza viruses in mammals and poultry. However, the host immune response and its role in disease severity and outcome of H5N6 AIVs in chickens and mice are unknown.

To investigate the origin, pathogenicity, transmission, and host immune response of the H5N6 AIVs in chickens and mice, we selected four H5N6 viruses, A/duck/Guangdong/13087/2013 (GD13087), A/goose/Guangdong/14016/2014 (GD14016), A/duck/Guangdong/14017/2014 (GD14017) and A/duck/Guangdong/14085/2014 (GD14085). These four H5N6 viruses are highly pathogenic in chickens, and can be transmitted between chickens by contact, but only cause mild to severe pathogenicity in mice.

## 2. Material and Methods

### 2.1. Viruses

The H5N6 viruses A/duck/Guangdong/13087/2013 (GD13087), A/goose/Guangdong/14016/2014 (GD14016), A/duck/Guangdong/14017/2014 (GD14017), and A/duck/Guangdong/14085/2014 (GD14085) were isolated from fecal samples of healthy white ducks and black mane geese in Guangdong, Southern China between 2013 and 2014. All of the isolated viruses were purified by three rounds of limiting dilution in 9- to 10-day-old specific-pathogen-free (SPF) embryonated chicken eggs. Aliquots of virus allantoic fluid stock were stored at –80 °C before use. Values of 50% embryo infective doses (EID_50_) and 50% embryo lethal doses (ELD_50_) were calculated by the Reed–Muench method [16].

Phylogenetic and sequence analysis. The eight genes of these viruses used in this study were sequenced. Viral RNA was extracted from allantoic fluid by using Trizol LS Reagent (Invitrogen Life Technologies, Inc. Carlsbad, CA, USA) and transcribed into cDNA with SuperScript III reverse transcriptase (Invitrogen Life Technologies, Inc. Carlsbad, CA, USA). PCR was performed using specific primers. The PCR products were purified with the QIAquick PCR purification kit (Qiagen, Hilden, Germany) and sequenced by Shanghai Invitrogen Biotechnology Co., Ltd. DNA sequences were compiled and edited using Lasergene 7.1 (DNASTAR, Madison, WI, USA). Phylogenetic trees were generated by the distance-based neighbor-joining method using MEGA 4.0 software (Sinauer Associates, Inc., Sunderland, MA, USA). The reliability of the tree was assessed by bootstrap analysis with 1000 replicates. Horizontal distances are proportional to genetic distance. The nucleotide sequences obtained in the present study are available from GenBank under the accession numbers (MN556606–MN556613, MN556678–MN556693, MN556722–MN556729).

### 2.2. Experimental Infection of Chickens

Twelve six-week-old SPF white leghorn chickens were inoculated intranasally with 0.1 mL allantoic fluid containing 10^6^EID_50_/0.1 mL of the GD13087, GD14016, GD14017, and GD14085 viruses, respectively. At 24 h post-infection, three contact chickens inoculated intranasally with 0.1 mL phosphate buffered saline (PBS) were housed together with the inoculated chickens. At 24 h post-infection, three infected chickens were euthanized for quantification of pattern-recognition receptors (PRRs) and cytokines in the lung and brain. At 3 days postinfection (DPI), three infected chickens were euthanized to test for the virus replication in different organs, including hearts, livers, spleens, lungs, kidneys, brains, tracheas, pancreases, intestines, and cloacal bursas. The remaining infected chickens in each group were observed for clinical symptoms for 14 days or until all the chickens were dead.

In addition, twelve chickens were not given any treatment as a control group. Three control chickens were euthanized at 24 h for quantification of pattern-recognition receptors (PRRs) and cytokines in the lung and brain; three control chickens at 4 days (that is to say: at 3 days postinfection in infected group) were euthanized for the virus detection in different organs; the remaining control chickens were used to observe for clinical symptoms for 14 days. Oropharyngeal and cloacal swabs were taken from the chickens at 1, 3, 5, 7, 9, 11 and 14 DPI, and suspended in 1 mL PBS. All of the tissues and swabs were collected and titrated for virus infectivity in embryonated chicken eggs as previously described [17]. Seroconversion of the surviving birds on 14 DPI was confirmed by hemagglutinin inhibition (HI) test.

### 2.3. Experimental Infection of Mice

Five- to six-week-old SPF female BALB/c mice were purchased from the Guangdong Medical Laboratory Animal Center in Guangzhou, China and housed in the isolators. To evaluate the mortality of the four viruses in mice, the mice were randomly divided into four groups of eleven mice each. After light anesthesia with CO_2_, the mice were inoculated intranasally with 10^6^EID_50_ of the virus in a 50 μL volume. Three mice in each infected group were euthanized at 3 and 5 DPI to determine virus titers in brain, spleen, kidney and lung, respectively, as previously described [18]. The remaining infected mice were monitored for weight loss and mortality for 14 days or until all the mice were dead. Additionally, eleven mice inoculated with 50 μL PBS served as negative controls. Three control mice were euthanized on days 4 and 6 for detection of the viruses in different organs. The remaining five control mice were monitored for weight loss and mortality for 14 days.

### 2.4. RNA Extraction and Quantitative Analysis of mRNA

RNA was prepared from 30 mg of lung and brain tissues from each chicken and mouse homogenized for 30 s twice in 600 μL buffer RLT Plus containing β-ME supplied in a Rneasy plus Mini Kit (Qiagen, Hilden, Germany). The next procedure followed the manufacturer’s instructions. The extracted RNA was eluted using 30 μL RNase-free water and the DNase-treated RNA (1 µg) was reverse transcribed onto complimentary DNA (cDNA) with the SuperScript III First Strand synthesis system (Invitrogen Life Technologies, Inc. Carlsbad, CA, USA). A quantitative real-time PCR (qRT-PCR) was performed using the QuantiFast SYBR Green PCR kit (Qiagen, Hilden, Germany). qRT-PCR was carried out in a 7500 Fast Real-Time PCR system by using the comparative threshold cycle (Ct) method to derive fold change gene expression, according to the manufacturer’s instructions (Applied Biosystems, Rotkreuz, Switzerland). The primers used for qRT-PCR were designed with Oligo7 software. The PCR conditions as follows: one cycle of 95 ℃ for 5 min followed by 40 cycles of 95 ℃ for 15 s and 60 ℃ for 34 s. Dissociation curves of the products were generated by increasing the temperature of samples incrementally from 55 to 100 ℃ as the final step of the PCR. The relative expression ratios of the target genes in the tested group versus those in the control group were calculated by the 2^−ΔΔCt^ method. Target gene Ct values were normalized to the endogenous control β-action, which is a house-keeping gene.

### 2.5. Statistical Analysis

Statistical analyses were conducted using the GraphPad Prism 5.0 software (GraphPad Software Inc., San Diego, CA, USA). The quantitative PRRs and cytokines of chickens and mice infected with the H5N6 viruses and control were compared using one-way ANOVA. Statistical analyses for virus titer in organs of control and infected chickens and mice were performed by using two-way ANOVA. The survival of chickens and mice were analyzed using log-rank (Mantel–Cox) tests and Gehan–Breslow–Wilcoxon tests. *p*-Values of <0.05 were considered significant.

### 2.6. Ethics Statements

All experiments were carried out in ABSL-3 facilities in compliance with the approved protocols of the biosafety committee of South China Agriculture University (SCAUABSL2017-001). The handling of chickens and mice were performed in accordance with the approved guidelines of the experimental animal administration and ethics committee of South China Agriculture University (SCAUABSL2017-001; 1 March, 2017).

## 3. Results

### 3.1. Phylogenetic Analysis of the H5N6 Viruses

To understand the genetic evolution of these H5N6 viruses, the eight genes of each virus were sequenced. The sequences were compared with representative sequences reported over the past decade in the GenBank database.

**Figure 1:**
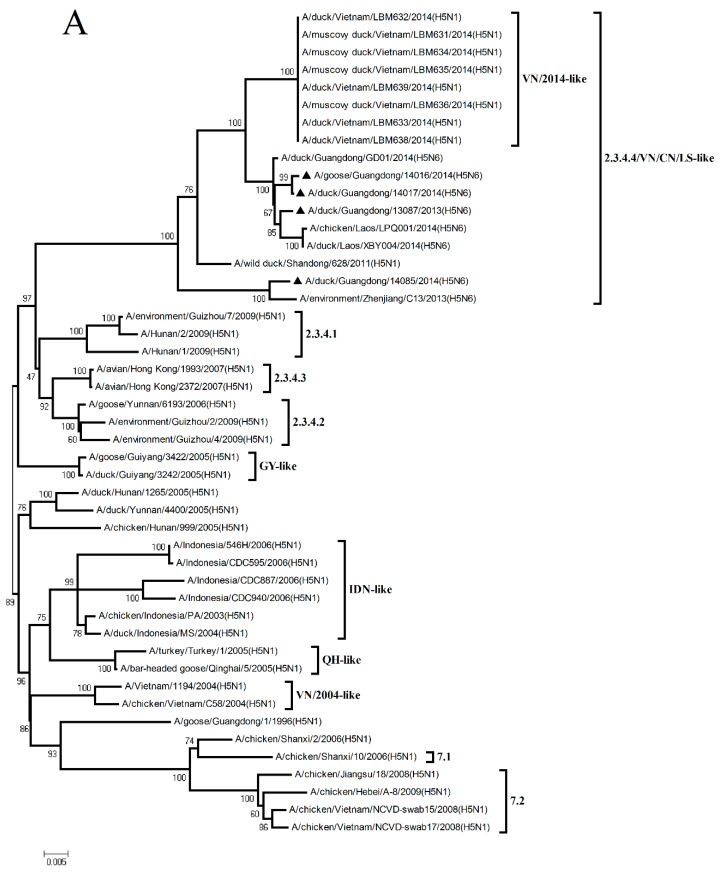

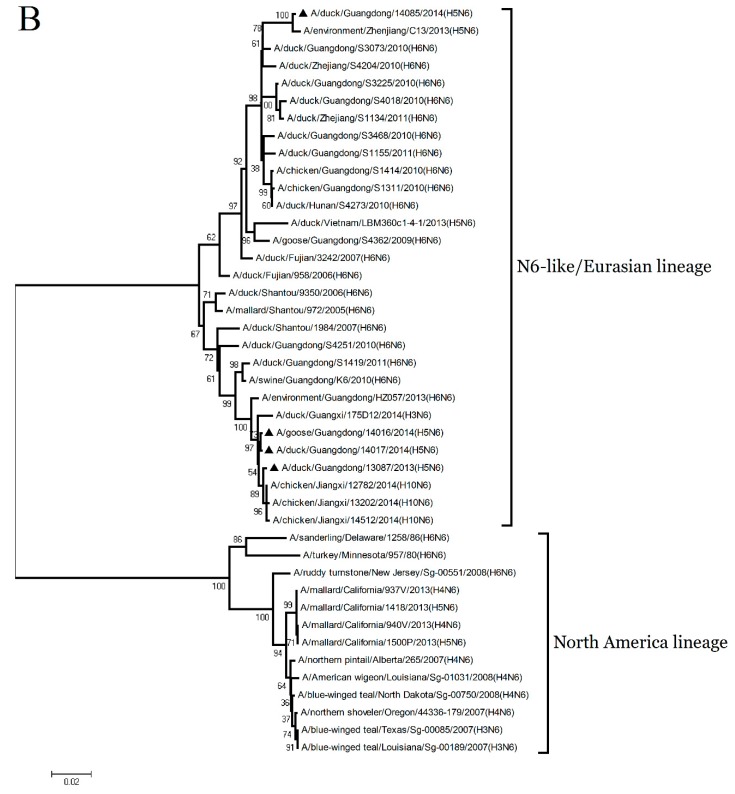
Phylogenetic analysis of HA and NA genes. The trees were constructed by using the neighbor-joining method with the maximum composite likelihood model and MEGA version 4.0 (http://www.megasoftware.net) with 1000 bootstrap replicates based on the following sequences: HA: nucleotides (nt) 29 to 1732 (**A**). NA: nucleotides (nt) 1 to 1413 (**B**). Abbreviation: IDN, Indonesia; QH, Qinghai; VN, Vietnam; GY, Guiyang. The virus marked with black triangle “▲” is the H5N6 virus isolated and sequenced in this study.

**Figure 2:**
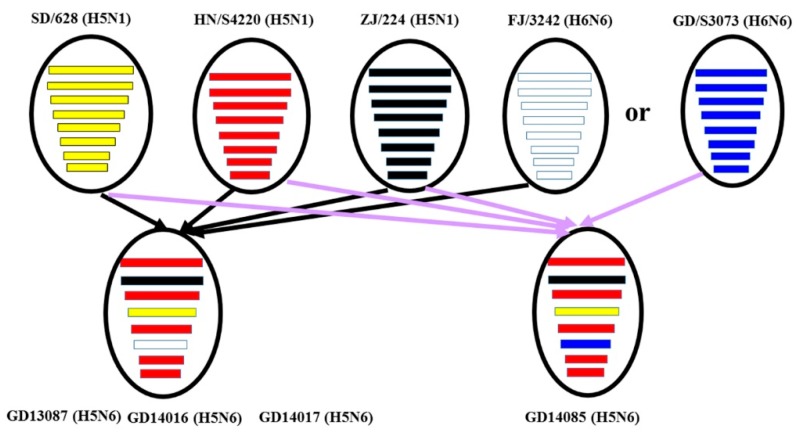
Genetic reassortant of the new H5N6 avian influenza viruses. The eight gene segments of four novel H5N6 viruses, represented by horizontal bars are, from top to bottom, PB2, PB1, PA, HA, NP, NA, M and NS. Each different color represents a distinct origin. Abbreviation: SD/628, A/wild duck/Shandong/628/2011(H5N1); HN/S4220, A/duck/Hunan/S4220/2011(H5N1); ZJ/224, A/duck/Zhejiang/224/2011(H5N1); FJ/3242, A/duck/Fujian/3242/2007(H6N6); GD/S3073, A/duck/Guangdong/S3073/2010(H6N6); GD13087, A/duck/Guangdong/13087/2013(H5N6); GD14016, A/goose/Guangdong/14016/2014(H5N6); GD14017, A/duck/Guangdong/14017/2014(H5N6); GD14085, A/duck/Guangdong/14085/2014 (H5N6).

The HA gene of the four H5N6 viruses shared nucleotide sequence similarities ranging from 97.1% to 97.4% with A/wild duck/Shandong/628/2011 (H5N1) (Figure 2 and Table S1). According to the antigenic characteristics reported by the WHO, the phylogenetic analysis of the HA gene showed that all of the H5N6 viruses in the present study belonged to Clade 2.3.4.4 and were clustered into the VN/CN/LS-like sublineage (Figure 1A). Thus, the phylogenetic analysis of these viruses indicated that the H5 variants have donated genes to generate the H5N6 viruses isolated in Southern China.

The NA genes of the GD13087, GD14016, and GD14017 viruses were closely related to A/duck/Fujian/3242/2007 (H6N6), with 92.4% to 92.6% nucleotide sequence similarities (Figure 2 and Supplementary Table S1). The NA gene of the GD14085 virus shared a high nucleotide sequence similarity (97.9%) with A/duck/Guangdong/S3073/2010 (H6N6) (Figure 2 and Supplementary Table S1). Phylogenetic analyses showed that the NA genes consist of two groups, the Eurasia lineage and the American lineage, and the four viruses fell into the N6-like sublineage/Eurasia lineage (Figure 1B). Thus, the phylogenetic analyses of these viruses indicated that the H6N6 variants isolated in Southern China have donated genes for the generation of the new H5N6 viruses and these H5N6 viruses discovered may become a potential source of novel pathogenic AIV strains such as the H10N6 [19].

The PB2, PA, NP, M, and NS genes of the GD13087, GD14016, GD14017, and GD14085 viruses were highly related to A/duck/Hunan/S4220/2011 (H5N1). The PB1 genes of the four viruses shared 98.1% to 98.3% nucleotide similarities with A/duck/Zhejiang/224/2011(H5N1) (Figure 2 and Supplementary Table S1). Phylogenetic analyses showed that the internal genes of the four viruses belonged to VN/CN/LS-like sublineage (Supplementary Figure S1). Thus, the phylogenetic analyses of these viruses indicate that the internal gene of the H5N1 viruses might be the progenitors of the H5N6 viruses.

### 3.2. Molecular Characterization

The HA genes of the four H5N6 isolates all had an open reading frame of 1704 bp, encoding 567 amino acids. The GD13087, GD14016, and GD14017 viruses contained the motif PLRERRRKR/GLF at the cleavage site between HA1 and HA2. The amino acid sequence of the cleavage site in the HA protein of the GD14085 virus was PLREKRRKR/GLF. These H5N6 viruses all had consecutive basic amino acids in the motif, thus meeting the characteristic for HPAIVs [20]. The amino acid residues in positions 190, 225, 226, 227, and 228 (H3 numbering is used) of these H5N6 viruses, which are well-established amino acid positions related to the receptor specificity of influenza viruses, were E190, G225, Q226, and G228, respectively; all of these viruses contained the avian-type motif. The mutation at position 227 (S227N) was proved to have a reduced binding affinity toward α-2, 3-linked sialic acid receptors and an increased affinity toward α-2, 6-linked sialic acid receptors. In our study, the four viruses carried an S227R mutation in the receptor-binding pocket of the HA protein. The amino acid sequences of the four viruses revealed five potential N-linked glycosylation sites in HA1 (26 or 27, 39, 181, and 302) and two in HA2 (499 and 558) (H5 numbering is used). The four viruses all exhibited de-glycosylation at residue 158 (residues 158–160: NDA) (H3 numbering is used) (Tables S2 and S3).

The NA genes of the GD13087, GD14016, and GD14017 viruses all had an open reading frame of 1380bp, encoding 459 amino acids. The NA gene of the GD14085 virus had an open reading frame of 1413bp, encoding 470 amino acids. The NA protein of the GD13087, GD14016, and GD14017 viruses had an eleven-amino-acid deletion of residues 58 to 68 in the stalk region, which is associated with increased virulence in mammals [21]. However, no deletion in the NA stalk region was found in the GD14085 virus. Mutations E119V, H275Y, R293K, and N295S were not detected in the NA protein of all the four viruses, suggesting that the isolates were sensitive to neuraminidase inhibitors [21]. The NA proteins of the GD13087, GD14016, and GD14017 viruses all had six potential N-glycosylation sites at positions 51, 54, 70, 86, 146, and 201. However, the N54S substitution removed the potential N-glycosylation sites at position 54 in the GD14085 virus; as a result, the GD14085 virus has only five potential N-glycosylation sites (Table S2 and Table S3).

Analysis of the internal genes of the four H5N6 viruses indicated that the lengths of the PB2, PB1, PA, NP, M, and NS genes were 2341, 2341, 2233, 1565, 1027, and 890 nucleotides, respectively. The six genes encode proteins with the following amino acid lengths: PB2, 759; PB1, 757; PA, 716; NP, 498; M1, 252; M2, 97; NS1, 225; and NS2, 121. The amino acid residue at position 627 of the PB2 protein of the four tested viruses was E rather than K, of which E627K is considered to be the predominant factor for the host range and the virulence of human HPAI H5N1 virus and HPAI H7N7 virus isolates [22]. The PB2 D701N is considered to increase the virulence, and to expand the host range of the avian H5N1 virus to mammalian hosts in the absence of E627K [22], but our four isolates were the Asparticacid (D) in this position. Single or multiple amino acid substitutions at positions L26F, V27A, A30T, S31N, or G34E in the transmembrane region of the M2 protein is the genetic basis for resistance to amantadine and rimantadine. No virus had amino acid substitutions at above-mentioned positions in the M2 protein, indicating that these viral strains are still sensitive to amantadine and rimantadine [23,24]. The four H5N6 viruses all had the sequence ESEV at the C terminus of the NS1 protein, which confers avian-like specificity [25]. The virulence of the influenza viruses in humans is related to their resistance to the antiviral effects of cytokines, such as interferon (IFN), and mutations D92E and P42S in the NS1 protein can increase resistance to IFN [26,27]. The H5N6 viruses in this study had mutations at residues 92 and 42 of the NS1 protein, respectively, suggesting these viruses may promote a greater resistance to these cytokines (Table S2 and Table S3).

### 3.3. Pathogenicity of the H5N6 Virus in Chickens

To investigate the pathogenicity of the H5N6 virus in chickens, we inoculated SPF chickens intranasally with the four viruses. Then, all the chickens were observed for 14 days after inoculation.

**Figure 3:**
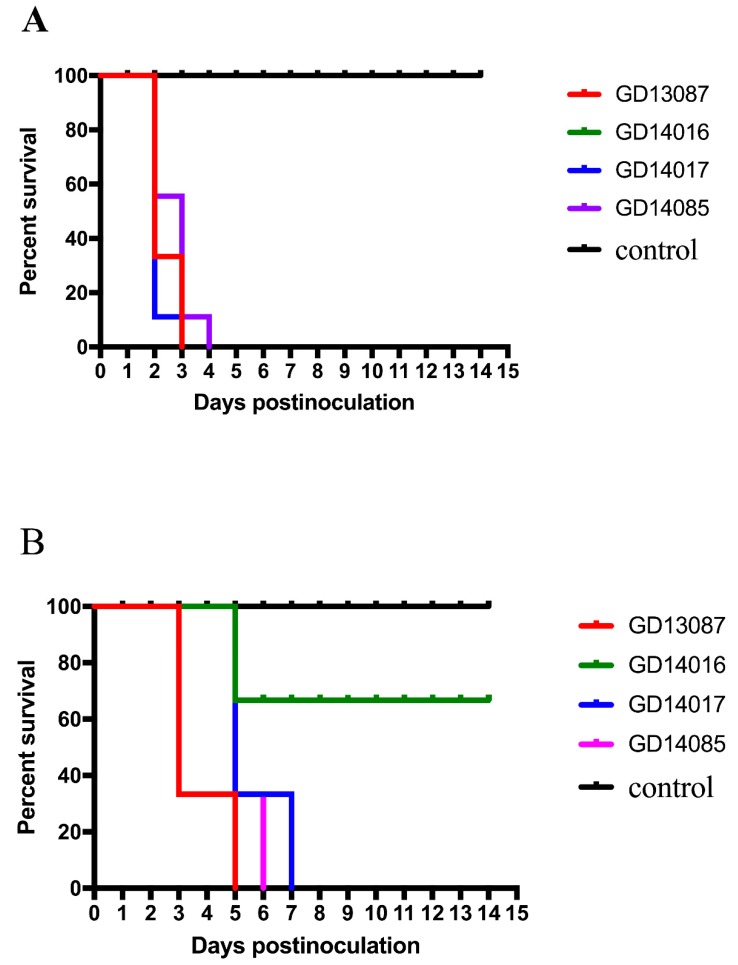
Lethality of the infected chickens (**A**) and contact chickens (**B**) in each group.

Most inoculated chickens in each group showed characteristic clinical symptoms including depression, reduction in food and water intake, ocular and nasal discharges, dyspnoea and/or conjunctivitis, incoordination and even nervous signs. The control chickens did not display any clinical symptoms and survived until the experiment finished. Compared to the control chickens, chickens inoculated with 10^6^EID_50_ of the GD13087, GD14016, GD14017, and GD14085 viruses experienced 100% (9/9) mortality within 3 and 4 DPI, respectively (*p* < 0.001; Figure 3A). Hence, these four viruses were highly pathogenic to chickens.

In the control chickens, no viruses were detected from all the tested organs. Compared to control chickens, the infected chickens in each group had the viruses in all the tested organs at 3 DPI, including the hearts, livers, spleens, lungs, kidneys, brains, tracheas, pancreases, intestines, and cloacal bursas (Table 1). The GD13087, GD14016, GD14017, and GD14085 viruses replicated to high titers in lung; the mean titers were 8.42, 8.33, 8.67, and 10.17 log_10_EID_50_, respectively. The four viruses could also replicate in the brains; the mean titers ranged from 7.33 to 8.75 log_10_EID_50_. Their mean titers were 8.67–9.50, 4.42–7.50, 5.75–7.50, 8.17–8.50, 3.33–6.50, 2.92–6.50, and 5.50–7.50 log_10_EID_50_ in the hearts, livers, spleens, kidneys, tracheas, pancreases, intestines, and cloacal bursas, respectively.

Additionally, shedding of the four H5N6 viruses from the inoculated chickens was detected in oropharyngeal and cloacal swabs at 1, 3, 5, 7, 9, 11, and 14 DPI (Table 2). At 1 DPI, eleven of the twelve (11/12) inoculated chickens could be detected shedding the GD13087 virus from oropharyngeal and cloacal swabs. All of the twelve chickens could be detected shedding the GD14016 virus from oropharyngeal swabs and eight of twelve chickens from cloacal swabs. The GD14017 virus was recovered from oropharyngeal swabs of all inoculated chickens, and from cloacal swabs of eight of the twelve chickens. All the chickens could be detected shedding the GD14085 virus from both oropharyngeal and cloacal swabs. At 3 DPI, the four viruses were recovered from oropharyngeal and cloacal swabs from all chickens inoculated. Therefore, the four viruses could replicate systemically, causing the death of all inoculated chickens, and shedding from all inoculated chickens.

To understand the transmission of these H5N6 viruses, three chickens housed with those chickens inoculated with the GD13087, GD14016, GD14017, and GD14085 viruses were inoculated intranasally with 0.1 mL phosphate buffered saline (PBS) as a naive contact group. During the observed period, some contact chickens showed clinical symptoms similar to those of the infected chickens. Compared to the contact chickens of the GD14016 virus, all the contact chickens of the GD13087, GD14017, and GD14085 viruses died within 5, 7, and 6 DPI (*p* < 0.01), respectively. The two survived contact chickens of the GD14016 virus did not seroconvert at the end of the experiment (Figure 3B).

Additionally, two-thirds (2/3) of the contact chickens could be detected shedding the GD13087 virus from oropharyngeal swabs and cloacal swabs at 1 DPI. The GD13087 virus could be detected in all contact chickens from oropharyngeal swabs and cloacal swabs at both 3 and 5 DPI. Two-thirds (2/3) of the contact chickens could be detected shedding the GD14016 virus from oropharyngeal swabs and only one contact chicken from cloacal swabs on 3 DPI. The GD14017 virus shedding could be detected from oropharyngeal swabs of all contact chickens at 3, 5, and 7 DPI. Two-thirds (2/3) of the contact chickens could be detected shedding the GD14085 virus from oropharyngeal swabs and cloacal swabs at 1 DPI. The GD14085 virus shedding could be detected from the oropharyngeal and cloacal swabs of all contact chickens at both 3 and 5 DPI (Table 2). Hence, the four viruses could be transmitted via direct contact and shed from the respiratory tract and the digestive tract of the contact chickens, but the four viruses had different capabilities for transmission.

Overall, our results indicated that these H5N6 viruses were highly pathogenic to chickens, and could be transmitted between chickens by contact, but had different capabilities for transmission.

### 3.4. Quantification of Pattern-Recognition Receptors (PRRs) and Cytokine in Chickens

**Figure 4:**
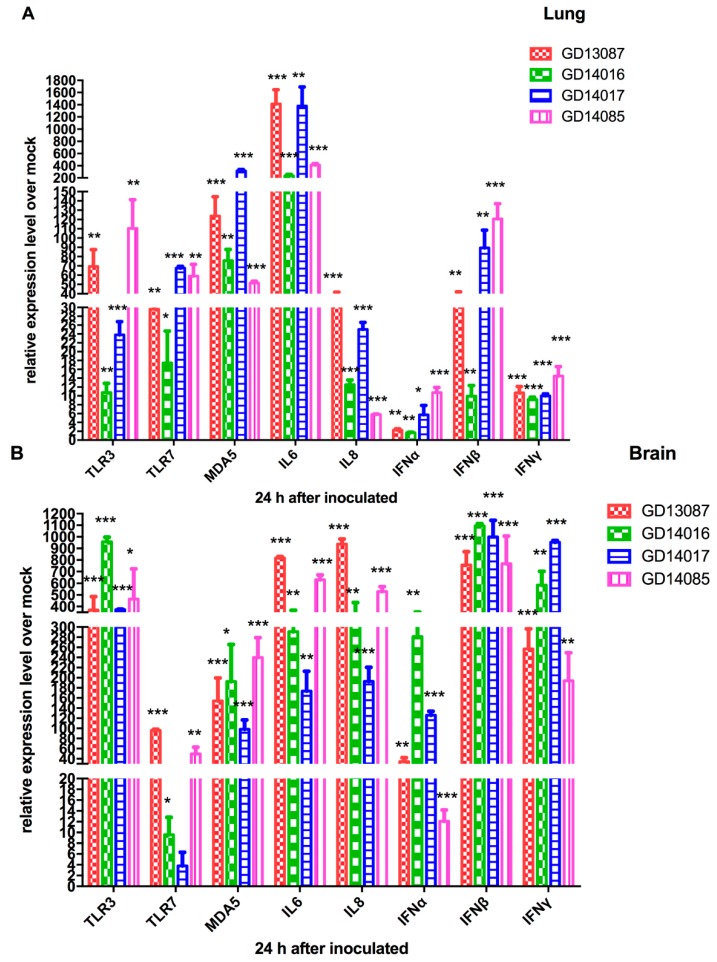
Pattern-recognition receptors (PRRs) and cytokine mRNA levels of chickens after infection by the H5N6 viruses. (**A**,**B**) PRRs and cytokine mRNA levels of chicken lung and brain after the GD13087, GD14016, GD14017, and GD14085 viruses challenge. Each bar represents the level of target gene mRNA relative to mock after normalizing data to the housekeeping gene β-action and presented as the mean values ± standard deviation (* *p* < 0.05, ** *p* < 0.01, *** *p* < 0.001). Error bars indicate standard deviations. Statistical analysis was performed using two-way ANOVA.

To determine the PRRs and cytokine levels following the infection of the H5N6 viruses at the early stage, we further tested the expression of TLR3, TLR7, MDA5 mRNA and a set of representative cytokines in the brains and lungs of chickens at 24 h postinfection using qRT-PCR (Figure 4A,B).

In the brains, TLR3 mRNA of all H5N6 virus-infected chickens were upregulated to a much greater degree (368- to 958-fold, *p* < 0.001) compared to that of control. TLR7 mRNA exhibited different levels of activation (4- to 96-fold) over that of control. The expression of MDA5 mRNA (98-to 240-fold; *p* < 0.01) was more highly activated by the four H5N6 viruses than that of control. In the lungs, TLR3 and TLR7 mRNA of all H5N6 virus-infected chickens exhibited different levels of activation (11- to 110-fold and 17- to 68-fold) over those of control. MDA5 mRNA of all H5N6 virus-infected chickens were significantly upregulated (317-fold, *p* < 0.001) compared to that of control.

In the brains, IL6 and IL8 mRNA, as pro-inflammatory cytokine genes, were significantly induced by the four viruses and exhibited 174- to 816-fold (*p* < 0.01) and 193- to 937-fold (*p* < 0.01) increases over those of control, respectively. IFNα mRNA induced by the four viruses exhibited different levels of activation (12- to 281-fold) over that of control, respectively. IFNβ and IFNγ mRNA of all H5N6 virus-infected chickens showed higher levels of activation compared to those of control (the GD13087 virus, 758-fold and 257-fold, *p* < 0.001 and *p* < 0.001, respectively; the GD14016 virus, 1094-fold and 584-fold, *p* < 0.001 and *p* < 0.001, respectively; the GD14017 virus, 999-fold and 952-fold, *p* < 0.001 and *p* < 0.001, respectively; the GD14085 virus, 768-fold and 194-fold, *p* < 0.001 and *p* < 0.001, respectively). In the lungs, IL6 mRNA induction by the four H5N6 viruses was more significant increase (234- to 1381-fold, *p* < 0.001, respectively) than that of control. IL8, IFNα, IFNβ, and IFNγ mRNA exhibited a wide range of activation after infection with these four H5N6 viruses.

### 3.5. Pathogenicity of the H5N6 Virus in BALB/c Mice

**Figure 5:**
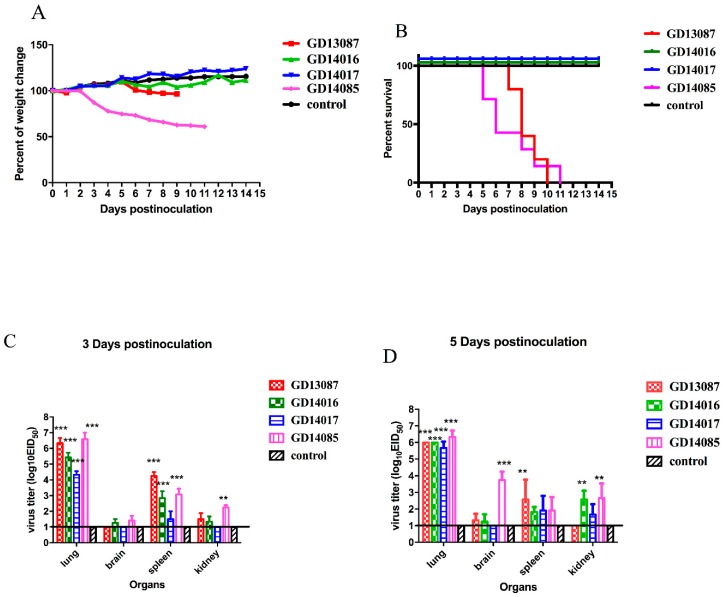
Weight change, lethality, and replication of the H5N6 viruses in mice. (**A**) Weight change of BALB/c mice during the 14 days postinoculation. Mice were inoculated intranasally with the doses of 10^6^EID_50_ of the H5N6 viruses. Mice inoculated with PBS served as a control group. (**B**) Lethality of the GD13087, GD14016, GD14017, and GD14085 viruses in BALB/c mice. Significant differences in survival were analyzed by log-rank (Mantel–Cox) tests and Gehan–Breslow–Wilcoxon tests. (**C**,**D**) Three six-week-old SPF BALB/c mice inoculated intranasally with 10^6^EID_50_ of each virus in a 50-μL volume were euthanized at 3 and 5 DPI and organs were collected for virus titration in eggs. For statistical analysis, a value of 1.0 was assigned if the virus was not detected from the undiluted sample in three embryonated hen eggs. Data shown are the mean virus titers ± standard deviation in log_10_EID_50_/g of tissue. Error bars indicate standard deviations. The straight line indicates the limit of detection. Statistical analysis was performed using two-way ANOVA.

The pathogenicity and pandemic potential of the viruses were next examined in mice, which are used as mammalian surrogates for humans in influenza research. SPF female BALB/c mice were inoculated with 10^6^EID_50_/50 μL. Three mice from each group were killed on days 3 and 5 after intranasal inoculation, and their organs were collected for virus titration in eggs. Compared to the mice in the control group, the GD13087 and GD14085 viruses caused a body weight reduction, and all mice died on 8 and 11 DPI, respectively (*p* < 0.01). The GD14017 virus caused an increase in body weight within the 14-day observation period (Figure 5A,B). All of the four avian-origin H5N6 viruses could replicate in mouse lungs compared to control mice (*p* < 0.001). The mean titers of the GD13087, GD14016, GD14017, and GD14085 viruses in the lungs reached 6.33, 5.42, 4.33, and 6.58log_10_ EID_50_, respectively, on 3 DPI and 6.00, 6.00, 5.67, and 6.33log_10_ EID_50_, respectively, on 5 DPI. To determine whether the H5N6 virus replicated systemically in the mice after intranasal infection, brains, spleens, and kidneys were also harvested from three infected mice from each group on 3 and 5 DPI. Compared to the control mice, the GD14085 and GD14016 viruses could be detected in the brains, spleens, and kidneys of the infected mice on both days. On 3 DPI, the GD13087 virus could be detected in the spleens (*p* < 0.001) and kidneys but not in the brains of the infected mice, and the GD14017 virus could be detected only in the spleens of the infected mice. On 5 DPI, the GD13087 virus could not be detected in kidneys. GD14017 did not spread to the brains on 5 DPI (Figure 5C,D).

These results revealed that the H5N6 viruses caused mild to severe pathogenicity and had different capability for replication in the mice. Therefore, these viruses may have different ability to infect mammals and may cause an epidemic in humans in the future.

### 3.6. Quantification of Pattern-Recognition Receptors (PRRs) and Cytokine in Mice

**Figure 6:**
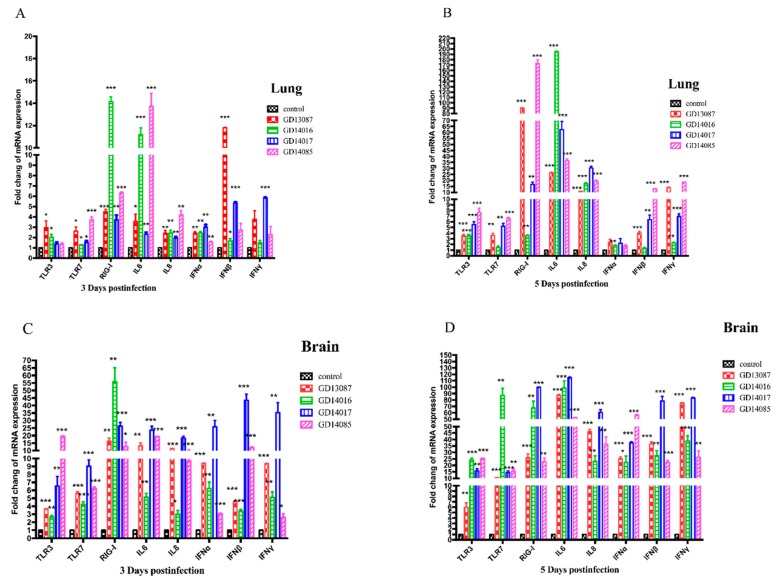
Pattern-recognition receptors (PRRs) and cytokine mRNA levels of mice after the H5N6 viruses’ infection. (**A**–**D**) PRRs and cytokine mRNA levels of mouse lung and brain after the GD13087, GD14016, GD14017, and GD14085 viruses challenge. Each bar represents the level of target gene mRNA relative to mock after normalizing data to the housekeeping gene β-action and presented as the mean values ± standard deviation (* *p* < 0.05, ** *p* < 0.01, *** *p* < 0.001). Error bars indicate standard deviations. Statistical analysis was performed using two-way ANOVA.

To determine the PRRs and cytokine levels following the infection of the H5N6 viruses at the early stage, we further measured the expression of the TLR3, TLR7, RIG-I mRNA, and a set of representative cytokines in the brains and lungs of mice at 3 and 5 DPI using qRT-PCR (Figure 6A–D).

In the brains, the expression of TLR3 and TLR7 mRNA for the four viruses were upregulated slightly at 3 DPI and then upregulated highly at 5 DPI over those of control, whereas RIG-I mRNA induction was more significantly increased (13- to 58-fold, *p* < 0.01) than those of control on both days. In the lungs, TLR3 and TLR7 mRNA of all H5N6 virus-infected chickens exhibited lower levels of activation on both days. The expression of RIG-I mRNA of the four H5N6 viruses was upregulated slightly at 3 DPI, (3- to 16-fold over that of control), but it was upregulated significantly at 5 DPI, (17- to 173-fold over those of control, *p* < 0.01).

In the brains, although IL6, IL8, IFNα, IFNβ and IFNγ mRNAs were significantly induced by these H5N6 viruses in comparison to those of the control at 3 DPI, they were induced to a much greater degree at 5 DPI (*p* *<* 0.001) than those of control. In the lungs, IL6 and IL8 mRNA were upregulated slightly at 3 DPI, but significantly increased compared to those of control (*p* *<* 0.05) at 5 DPI. IFNα, IFNβ, and IFNγ mRNA exhibited lower levels of activation on both days.

## 4. Discussion

The H5N6 HPAIV in clade 2.3.4.4 have emerged and caused human infections and important HPAIV outbreaks of different bird species in China, Denmark, Finland, Germany, Greece, Iran, Japan, Korea, Laos, Myanmar, Netherlands, Philippines, Slovakia, Sweden, Switzerland, United Kingdom, and Vietnam in the recent period of time. From 2014 to 2019, about 66 outbreaks of the H5N6 HPAIV, including 226,794 cases and 169,027 deaths in birds, have been documented in China and caused serious losses to the poultry industry [28]. In our study, we isolated four H5N6 HPAI viruses from cloacal swabs of waterfowl in live bird markets during 2013 and 2014. All the four viruses belonged to the VN/CN/LS -like lineage, which originated from the Gs/GD lineage. The HA genes of the viruses clustered in 2.3.4.4 and shared the highest nucleotide sequence similarity with A/wild duck/Shandong/628/2011 (H5N1), which might be the progenitor of these H5N6 viruses [29]. The NA genes of these viruses were derived from H6 subtype viruses, which have crossed the species barrier and infected mammals, even humans, without adaptation in recent years [21]. The six internal genes of the viruses were derived from avian H5N1 viruses (A/duck/Hunan/S4220/2011 (H5N1) and/or A/duck/Zhejiang/224/2011(H5N1)). The results of the genetic analyses suggested that the four H5N6 AIVs may be multi-reassortant among different genotypes of avian influenza viruses. In China, live bird markets provide mixing grounds for different poultry species infected with different avian influenza viruses. These viruses carried by incoming and/or unsold birds continue to circulate and amplify within these markets. So live bird markets are usually considered to be an ideal environment for genetic reassortment and interspecies transmission of influenza viruses [30]. Therefore, active surveillance of live bird markets is important for understanding the evolution and antigen variation of H5 avian influenza viruses in China.

The influenza virus life cycle is initiated by the binding of viral HA to receptors on host cells. Usually, influenza A viruses circulating in wild waterfowl and domestic birds efficiently bind to α-2, 3-linked sialic acid receptors, whereas those of human viruses are adapted to efficiently bind to α-2, 6-linked sialic acid receptors. The earliest human H2 and H3 isolates differed from their avian ancestor, those isolates preferred binding to α-2, 6-linked sialic acid receptors rather than α-2, 3-linked sialic acid receptors, suggesting that a shift from avian virus α-2, 3-linked sialic acid receptors to a human virus α-2, 6-linked sialic acid receptors is critical for efficient replication and spread of a novel influenza strain in humans [31]. However, the H7N9 avian influenza virus isolated from humans in 2013, which possesses all eight gene segments from avian influenza viruses, still strongly interacts with α-2, 3-linked sialic acid receptors. It is worth noting that these H7N9 viruses gained the low levels of binding to α-2, 6-linked sialic acid receptors after transmission from birds to humans [32]. The S227N change associated with de-glycosylation at residue 158 (H3 numbering is used) in H5N1 influenza virus increased binding to a α-2, 6-linked sialic acid receptor and retained binding to α-2, 3-linked sialic acid receptors [33]. De-glycosylation at residue 158 was also critical for the transmission of H5N1 viruses in guinea pigs [34]. Additionally, the H5N6 viruses in our study have mutations S227R and de-glycosylation at residue 158. Therefore, we should also pay attention to whether this S227R affects the receptor-binding property.

The pathogenicity of avian influenza viruses often depends on whether they can replicate systematically and the viral loads in organs. Until now, the H5N6 avian influenza viruses have been isolated from humans, wild birds, domestic poultry, cats, and swine in China [9,10,35,36,37,38]. Further, compared to previous studies about H5N6 HPAI viruses, these viruses show altered pathogenicity in birds and mammals. Wu and colleagues have demonstrated that the H5N6 avian viruses isolated from poultry were highly pathogenic in chickens and moderately virulent in mice [36]. The H5N6 viruses isolated from apparently healthy pigs were highly pathogenic to chickens and replicated well in trachea, lung, spleen, kidney, pancreas, brain and cloaca of chickens, but could only replicate in the mouse lung, causing bronchopneumonia symptoms [37]. In our study, the four H5N6 avian influenza viruses from waterfowl caused 100% (9/9) mortality in the inoculated chickens within 4 DPI. Unlike previous studies, the H5N6 viruses in our study could not only replicate systemically but also transmit between the chickens via contact. Some of them could even result in the death of the mice, replicate systemically with different viral loads, and even spread to the brain through the blood–brain barrier. Therefore, our results demonstrated that the four H5N6 avian influenza viruses were highly pathogenic to the chickens and transmitted among the chickens, but only had mild to severe pathogenicity in mice.

The molecular basis of the pathogenicity of H5N6 HPAI viruses in chickens and mice remains to be determined. Several critical amino acids contributing to the pathogenicity of HPAI viruses in animals have been identified. All of the four H5N6 viruses in this study had multiple basic amino acids at the hemagglutinin (HA) cleavage site, meeting the characteristic for HPAI viruses. All of the four H5N6 viruses had glutamic acid and aspartic acid at positions 627 and 701 in PB2, respectively. The serine and glutamic acid at positions 42 and 92 in the NS1 protein of the four viruses confers resistance to antiviral cytokines in mice and pigs. Studies have demonstrated that amino acid deletion in the NA stalk region is associated with increased virulence in mammals [21]. GD13087, GD14016, and GD14017 viruses in this study have an 11-amino-acid deletion in the NA stalk region, but the GD13087 virus caused lethal infection in mice and the GD14016 and GD14017 viruses only cause mild infection in mice. GD14085 virus does not have such deletion, otherwise, GD14085 caused lethal infection like GD13087 virus in mice. This result indicated that there may be other mutations in the NA gene or other genes of the virus may also contribute to the virulence in mammals. In addition, whole-genome-sequence comparisons revealed that tens of amino acids in eight gene segments of the four viruses were different from each other (Table S4). Therefore, these different amino acids among the four viruses may result in the various pathogenicity in mice.

Besides systemic replication and high viral loads, the pathogenicity of the influenza virus is also dependent on the host immune response. The innate immune response is the first line of defense and limits virus replication. Viral endocytosis is usually followed by activation of intracellular pattern recognition receptors, which include TLR3, TLR7, RIG-I, MDA5 and NOD2 [39,40]. These receptors triggered by the influenza virus lead to a downstream signaling cascade that results in the activation of the transcription factors NF-κβ and IRF3 and the secretion of pro-inflammatory cytokines and type I IFNs. Elevated cytokine levels recruit lymphocytes to the target organ and facilitate the reduction of the initial viral replication and viral spread. However, an inordinate or imbalanced immune response may result in an overproduction of cytokines as well as chemokines that can lead to severe inflammation, including excessive recruitment of neutrophils and mononuclear cells at the site of infection. The inflammatory tissue damage results in poor clinical outcomes and pathogenesis during influenza virus infection in humans and animal models [41,42]. Previous studies have showed that H5N1 HPAIV infections in humans that caused death were associated with high viral loads and hyper-cytokinemia [43]. Cytokine dysregulation in H5N1 pathogenesis was also discovered in vitro and in animal experiments, which was characterized by the evasion of the antiviral effects of interferons, tumor necrosis factor (TNF)-α, and pro-inflammatory cytokines [43]. Similarly, H7N9-infected humans and animals are capable of eliciting elevated levels of pro-inflammatory cytokine (such as IL6, IL8) [44,45]. In our study, the four H5N6 avian influenza viruses induced significant up-regulation of PRR signaling in lungs and brains of chickens (TLR3 and MDA5) and mice (TLR3 and RIG-I), and elevated levels of pro-inflammatory cytokines (IL6 and IL8) in brains and lungs of chickens and mice. More interesting, we found that GD14016 and GD14017 viruses, with mild pathogenicity to mice, induced upregulation of most cytokines tested to levels comparable or even higher than those induced by GD13087 and GD14085 viruses in the brain tissues of mice on 5 DPI. Yu et al. found that the mRNA levels of TNF-α, IL-6, and IL-1β of the brains of mice infected with the 10^3^TCID_50_/mL A/NWS/33(H1N1) virus showed a statistically significantly higher level than that in control brains at 5 DPI. However, the mice infected with 10^3^TCID_50_/mL of the influenza virus survived until the experiment ended [46]. Therefore, we suggest that the levels of cytokines induced by influenza viruses in host might be not completely related to their pathogenicity.

In summary, the new H5N6 avian influenza viruses are highly pathogenic to chickens and transmitted among chickens, but have different pathogenicity in mice. The high viral loads of the H5N6 viruses in several organs resulted in disease severity in chickens and mice, and there were varying levels of cytokines induced by the H5N6 viruses with different pathogenicity in chickens and mice.

## Figures and Tables

**Table 1:** Replication in the chickens of the H5N6 virus after inoculated intranasally ^a^.

Strains	Virus Stock Titer (log_10_EID_50_)	Virus Replication on 3 DPI (log_10_EID_50_/0.1 mL) ^b^ in									
		Heart	Liver	Spleen	Lung	Kidney	Brain	Trachea	Pancreas	Intestine	Cloacal Bursa
GD13087	8.50	9.50 ± 0	7.50 ± 0	7.50 ± 0	8.42 ± 0.14	8.50 ± 0	7.58 ± 0.29	6.42 ± 0.14	6.50 ± 0	6.92 ± 1.01	7.50 ± 0
GD14016	8.63	8.67 ± 1.13	7.17 ± 0.58	7.42 ± 0.14	8.33 ± 0.72	8.17 ± 0.58	7.33 ± 1.01	6.50 ± 0	6.50 ± 0	7.25 ± 0.43	7.42 ± 0.14
GD14017	9.25	9.33 ± 0.14	7.50 ± 0	7.50 ± 0	8.67 ± 0.52	8.17 ± 0.58	7.67 ± 0.88	6.42 ± 0.14	6.50 ± 0	7.50 ± 0	6.83 ± 1.16
GD14085	8.38	9.42 ± 1.13	4.42 ± 1.66	5.75 ± 1.15	10.17 ± 0.14	8.17 ± 0.38	8.75 ± 1.56	3.33 ± 1.23	2.92 ± 2.24	4.50 ± 1.73	5.50 ± 0

^a^ Six-week-old SPF chickens were inoculated intranasally (i.n.) with 10^6^EID_50_ of the GD13087, GD14016, GD14017, and GD14085 viruses in a volume of 0.1 mL; three chickens were chosen for virus titer on 3 DPI in each group (except for the group infected with the GD14017 virus, because only one chicken was left, in order to meet the statistical significance, two chickens that died on 2 DPI were chosen), and the heart, liver, spleen, lung, kidney, brain, trachea, pancreas, intestine, and cloacal bursa of the three chosen chickens were collected for virus titer in eggs. ^b^ For statistical analysis, a value of 1.5 was assigned if the virus was not detected from the undiluted sample in three embryonated chicken eggs. Virus titers are expressed as means ± standard deviation in log_10_EID_50_/0.1 mL of tissue.

**Table 2:** Virus shedding in oropharyngeal and cloacal swabs from inoculated and contacted chickens.

Strain	Infection Sample	1 DPI		3 DPI		5 DPI		7 DPI		9 DPI		11 DPI		14 DPI	
		T	C	T	C	T	C	T	C	T	C	T	C	T	C
GD13087	*Inoculated*	*11/12* ^a^	*11/12*	*3/3*	*3/3*	- ^b^	-	-	-	-	-	-	-	-	-
	*Contacted*	*2/3*	*2/3*	*3/3*	*3/3*	*1/1*	*1/1*	-	-	-	-	-	-	-	-
GD14016	*Inoculated*	*12/12*	*8/12*	*3/3*	*3/3*	-	-	-	-	-	-	-	-	-	-
	*Contacted*	*0/3*	*0/3*	*2/3*	*1/3*	*1/3*	*1/3*	*0/2*	*0/2*	*0/2*	*0/2*	*0/2*	*0/2*	*0/2*	*0/2*
GD14017	*Inoculated*	*12/12*	*8/12*	*1/1*	*1/1*	-	-	-	-	-	-	-	-	-	-
	*Contacted*	*0/3*	*0/3*	*3/3*	*1/3*	*3/3*	*3/3*	*1/1*	*1/1*	-	-	-	-	-	-
GD14085	*Inoculated*	*12/12*	*12/12*	*5/5*	*5/5*	-	-	-	-	-	-	-	-	-	-
	*Contacted*	*2/3*	*2/3*	*3/3*	*3/3*	*3/3*	*3/3*	-	-	-	-	-	-	-	-

Abbreviations: DPI, day post-inoculation; T, oropharyngeal swab; C, cloacal swab. ^a^ virus-positive birds/tested birds. ^b^ all of the chickens died during the observation.

## Supplementary Materials

The following are available online at https://www.mdpi.com/1999-4915/11/11/1048/s1, Figure S1: Phylogenetic analysis of PB2, PB1, PA, NP, M1, NS1, Table S1: Sequence comparisons of the four H5N6 avian influenza viruses with their Closest genetic relatives, Table S2: Summary of Signature Amino Acid Mutation in GD13087, GD14016, GD14017, and GD14085 Viruses, Table S3: Summary of the Potential glycosylation site in HA and NA genes of the GD13087, GD14016, GD14017, and GD14085 viruses, Table S4: Amino-acid Difference among the GD13087, GD14016, GD14017, and GD14085 viruses.
