# Supplementary material for: Phylogeny, Pathogenicity, Transmission, and Host Immune Responses of Four H5N6 Avian Influenza Viruses in Chickens and Mice
Source: Viruses. 2019 Nov 10;11(11):1048. doi: 10.3390/v11111048 (PMC6893672; doi:10.3390/v11111048)
Supplement: Supplementary file 1 [file viruses-11-01048-s001.pdf]

Phylogeny, Pathogenicity, Transmission and Host Immune Responses of New H5N6 Avian Influenza Viruses in Chickens and Mice

Yafen Song[1,2], Weiqiang Li [1], Wenbo Wu [1], Zhiting Liu[1], Zhuoliang He[1], Zuxian Chen[1], Bingbing Zhao[1], Siyu Wu[1], Chenghuai Yang[2], Xiaoyun Qu[1], Ming Liao[1*], Peirong Jiao[1*]

[1] College of Veterinary Medicine, South China Agricultural University, Guangzhou 510642, China

[2] China Institute of Veterinary Drug Control, Beijing 100081, China

* Correspondence: prjiao@scau.edu.cn and mliao@scau.edu.cn; Tel.: (+86) 020-85283309

Supplemental material: Table S1-S4 and Fig. S1.

Table S1. Sequence comparisons of the four H5N6 avian influenza viruses with their Closest genetic relatives

Virus	Gene	Closest related virus strain	Homology (%)
A/duck/Guangdong/13087/2013 (GD13087)	PB2	A/duck/Hunan/S4220/2011(H5N1)	98.6
	PB1	A/duck/Zhejiang/224/2011(H5N1)	98.3
	PA	A/duck/Hunan/S4220/2011(H5N1)	99.1
	HA	A/wild/duck/Shandong/628/2011(H5N1)	97.4
	NP	A/duck/Hunan/S4220/2011(H5N1)	98.5
	NA	A/duck/Fujian/3242/2007(H6N6)	92.4
	M	A/duck/Hunan/S4220/2011(H5N1)	99.2
	NS	A/duck/Hunan/S4220/2011(H5N1)	98.9
A/goose/Guangdong/14016/2014 (GD14016)	PB2	A/duck/Hunan/S4220/2011(H5N1)	98.6
	PB1	A/duck/Zhejiang/224/2011(H5N1)	98.2
	PA	A/duck/Hunan/S4220/2011(H5N1)	99.0
	HA	A/wild duck/Shandong/628/2011(H5N1)	97.3
	NP	A/duck/Hunan/S4220/2011(H5N1)	98.5
	NA	A/duck/Fujian/3242/2007(H6N6)	92.6
	M	A/duck/Hunan/S4220/2011(H5N1)	99.2
	NS	A/duck/Hunan/S4220/2011(H5N1)	99.0
A/duck/Guangdong/14017/2014 (GD14017)	PB2	A/duck/Hunan/S4220/2011(H5N1)	98.6
	PB1	A/duck/Zhejiang/224/2011(H5N1)	98.2
	PA	A/duck/Hunan/S4220/2011(H5N1)	99.0
	HA	A/wild duck/Shandong/628/2011(H5N1)	97.4
	NP	A/duck/Hunan/S4220/2011(H5N1)	98.5
	NA	A/duck/Fujian/3242/2007(H6N6)	92.6
	M	A/duck/Hunan/S4220/2011(H5N1)	99.1
	NS	A/duck/Hunan/S4220/2011(H5N1)	99.0
A/duck/Guangdong/14085/2014 (GD14085)	PB2	A/duck/Hunan/S4220/2011(H5N1)	98.3
	PB1	A/duck/Zhejiang/224/2011(H5N1)	98.1
	PA	A/duck/Hunan/S4220/2011(H5N1)	98.9
	HA	A/wild duck/Shandong/628/2011(H5N1)	97.1
	NP	A/duck/Hunan/S4220/2011(H5N1)	98.3
	NA	A/duck/Guangdong/s3073/2010(H6N6)	97.9
	M	A/duck/Hunan/S4220/2011(H5N1)	98.9
	NS	A/duck/Hunan/S4220/2011(H5N1)	99.3

Table S2. Summary of Signature Amino Acid Mutation in GD13087, GD14016, GD14017, and GD14085 Viruses.

Gene	Amino Acid position	Virus			
		GD13087	GD14016	GD14017	GD14085
HA[a]	190	E	E	E	E
	225	G	G	G	G
	226	Q	Q	Q	Q
	227	R	R	R	R
	228	G	G	G	G
	325-336 (Cleavage site)	PLRERRRKRGLF	PLRERRRKRGLF	PLRERRRKRGLF	PLREKRRKRGLF
NA[b]	58-68 (Stalk region)	deletion	deletion	deletion	TIINNHPQNNF
	119	E	E	E	E
	275	H	H	H	H
	293	R	R	R	R
	295	N	N	N	N
PB2[c]	627	E	E	E	E
	701	D	D	D	D
M2[c]	26	L	L	L	L
	27	V	V	V	V
	30	A	A	A	A
	31	S	S	S	S
	34	G	G	G	G
NS1[c]	42	S	S	S	S
	92	E	E	E	E

[a] According to H3 numbering.
[b] According to N6 numbering.
[c] According to H5 numbering.

Table S3. Summary of the Potential glycosylation site in HA and NA genes of the GD13087, GD14016, GD14017, and GD14085 viruses.

Segments	Amino Acid position[a]	Virus			
		GD13087	GD14016	GD14017	GD14085
HA1	26	NNS	NNS	NNS	NNS
	27	NST	NST	NST	NST
	39	NVT	NVT	NVT	NVT
	181	NNT	NNT	NNT	NNT
	302	NSS	NSS	NSS	NSS
HA2	499	NGT	NGT	NGT	NGT
	558	NGS	NGS	NGS	NGS
NA	51	NET	NET	NET	NET
	54	NPT	NPT	NPT	-[b]
	70	NIT	NIT	NIT	NIT
	86	NLT	NLT	NLT	NLT
	146	NGT	NGT	NGT	NGT
	201	NAS	NAS	NAS	NAS

[a] Amino acids sites (HA segment and NA segment) were located by taking A/goose/Guangdong/1/1996 (H5N1), A/duck/Guangdong/s3073/2010(H6N6) and A/duck/Fujian/3242/2007(H6N6).

[b] The potential N-glycosylation sites at position is absent.

Table S4. Amino-acid Difference among the GD13087, GD14016, GD14017, and GD14085 viruses.

Gene	Amino Acid position	Virus			
		GD13087	GD14016	GD14017	GD14085
HA[a]	10	V	V	V	I
	16	S	S	S	G
	46	Q	Q	Q	R
	56	R	R	R	K
	74	G	G	G	G
	110	N	S	S	N
	130	T	T	T	I
	139	P	P	P	T
	142	E	E	E	D
	156	M	M	M	T
	167	T	T	T	I
	177	E	K	K	K
	178	M	M	M	I
	185	R	R	R	Q
	214	V	V	V	I
	261	G	E	G	G
	263	F	Y	F	F
	285	M	M	M	V
	341	R	R	R	K
	383	R	R	R	K
	393	I	V	V	V
	516	K	E	E	E
NA[b]	15	V	V	V	M
	22	E	E	E	E
	39	L	L	L	M
	44	T	T	T	N
	54	N	N	N	T
	58-68 (Stalk region)	deletion	deletion	deletion	TIINNHPQNNF
	77	N	N	N	T
	82	R	R	R	G
	83	T	T	T	H
	115	L	L	L	I
	143	R	R	R	K
	159	I	I	I	V
	209	R	R	R	G
	220	V	A	A	A
	235	K	K	K	G

Table S4. Amino-acid Difference among the GD13087, GD14016, GD14017, and GD14085 viruses. Continued

Gene	Amino Acid position	Virus			
		GD13087	GD14016	GD14017	GD14085
NA[b]	250	K	K	K	R
	252	A	A	A	E
	264	Q	Q	Q	K
	270	Q	Q	Q	K
	272	N	N	N	D
	285	A	A	A	S
	286	G	G	G	E
	291	V	V	V	I
	303	I	I	I	V
	316	K	K	K	R
	338	D	D	D	E
	391	P	P	P	A
	392	T	T	T	I
	394	Y	Y	Y	H
	396	L	L	L	I
	416	N	T	T	N
	436	I	S	S	S
	437	D	D	D	S
	445	M	M	M	I
	452	R	R	R	K
PB2[a]	144	R	R	R	Q
	299	K	K	K	R
	451	I	I	I	L
	637	I	I	I	T
	754	I	I	I	N
PB1[a]	386	K	K	K	R
	387	K	K	K	E
	430	R	R	R	K
	621	Q	Q	Q	K
	635	R	R	R	K
	648	A	A	A	T
	654	G	G	G	S
PA[a]	254	N	N	N	D
	261	M	M	M	I
	262	K	K	K	R
	272	D	D	D	E
	300	E	E	E	K
	405	S	S	S	G
	677	E	G	G	E
	688	D	D	D	E

Table S4. Amino-acid Difference among the GD13087, GD14016, GD14017, and GD14085 viruses. Continued

Gene	Amino Acid position	Virus			
		GD13087	GD14016	GD14017	GD14085
NP[a]	27	A	T	T	A
	33	V	V	V	I
	105	I	I	I	M
	186	V	V	V	I
	450	N	N	N	S
M1[a]	37	A	T	A	T
	75	Q	L	Q	Q
	116	A	S	A	S
M2[a]	13	N	N	N	K
	50	Y	C	Y	C
	97	E	E	E	K
NS1[a]	18	I	V	V	V
	122	S	S	S	T
	192	I	I	I	V

[a] According to H5 numbering.

[b] According to N6 numbering.

Fig S1. Phylogenetic analysis of PB2, PB1, PA, NP, M1, NS1. The trees were constructed by using the neighbor joining method with the Maximum Composite Likelihood model and MEGA version 4.0 **(**http://www.megasoftware.net**)** with 1,000 bootstrap replicates based on the following sequences: PB2 (S1A): nt 28 to 2307; PB1 (S1B): nt 25 to 2298; PA (S1C): nt 25 to 2175; NP (S1D): nt 46 to 1542; M1 (S1E): nt 26 to 784; NS1 (S1F): nt 27 to 704. Abbreviation: IDN, Indonesia; QH, Qinghai; VN, Vietnam; GY, Guiyang. The virus marked with black triangle '▲'is the H5N6 virus isolated and sequenced in this study.

Fig. S1A.

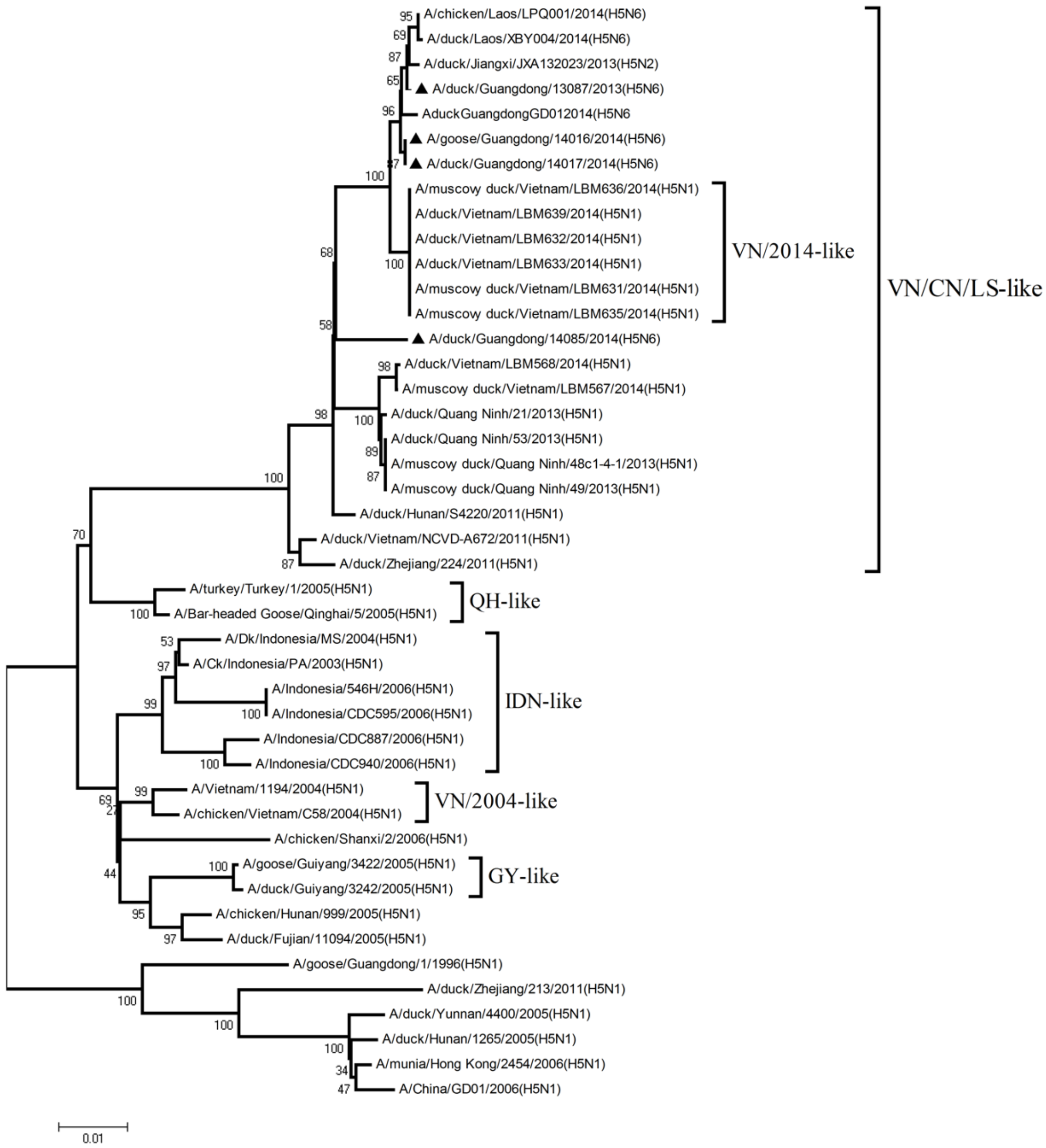

Fig. S1B.

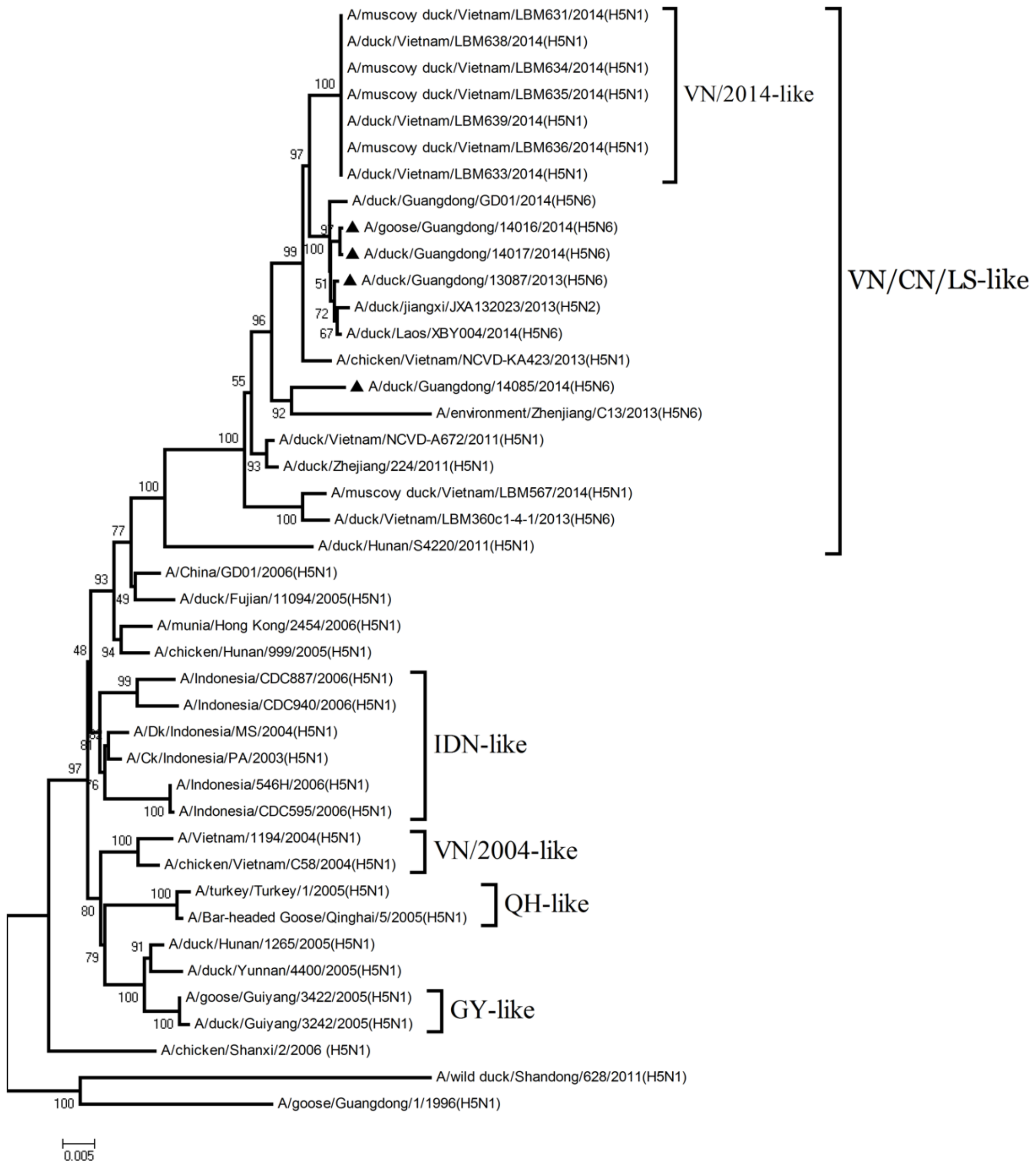
A/muscovy duck/Vietnam/LBM631/2014(H5N1)
A/duck/Vietnam/LBM638/2014(H5N1)
A/muscovy duck/Vietnam/LBM634/2014(H5N1)
A/muscovy duck/Vietnam/LBM635/2014(H5N1)
A/duck/Vietnam/LBM639/2014(H5N1)
A/muscovy duck/Vietnam/LBM636/2014(H5N1)
A/duck/Vietnam/LBM633/2014(H5N1)
VN/2014-like
A/duck/Guangdong/GD01/2014(H5N6)
▲ A/goose/Guangdong/14016/2014(H5N6)
▲ A/duck/Guangdong/14017/2014(H5N6)
▲ A/duck/Guangdong/13087/2013(H5N6)
A/duck/jiangxi/JXA132023/2013(H5N2)
A/duck/Laos/XBY004/2014(H5N6)
A/chicken/Vietnam/NCVD-KA423/2013(H5N1)
▲ A/duck/Guangdong/14085/2014(H5N6)
A/environment/Zhenjiang/C13/2013(H5N6)
A/duck/Vietnam/NCVD-A672/2011(H5N1)
A/duck/Zhejiang/224/2011(H5N1)
A/muscovy duck/Vietnam/LBM567/2014(H5N1)
A/duck/Vietnam/LBM360c1-4-1/2013(H5N6)
A/duck/Hunan/S4220/2011(H5N1)
VN/CN/LS-like
A/China/GD01/2006(H5N1)
A/duck/Fujian/11094/2005(H5N1)
A/munia/Hong Kong/2454/2006(H5N1)
A/chicken/Hunan/999/2005(H5N1)
A/Indonesia/CDC887/2006(H5N1)
A/Indonesia/CDC940/2006(H5N1)
A/Dk/Indonesia/MS/2004(H5N1)
A/Ck/Indonesia/PA/2003(H5N1)
A/Indonesia/546H/2006(H5N1)
A/Indonesia/CDC595/2006(H5N1)
IDN-like
A/Vietnam/1194/2004(H5N1)
A/chicken/Vietnam/C58/2004(H5N1)
VN/2004-like
A/turkey/Turkey/1/2005(H5N1)
A/Bar-headed Goose/Qinghai/5/2005(H5N1)
QH-like
A/duck/Hunan/1265/2005(H5N1)
A/duck/Yunnan/4400/2005(H5N1)
A/goose/Guiyang/3422/2005(H5N1)
A/duck/Guiyang/3242/2005(H5N1)
GY-like
A/chicken/Shanxi/2/2006 (H5N1)
A/wild duck/Shandong/628/2011(H5N1)
A/goose/Guangdong/1/1996(H5N1)
0.005

Fig. S1C.

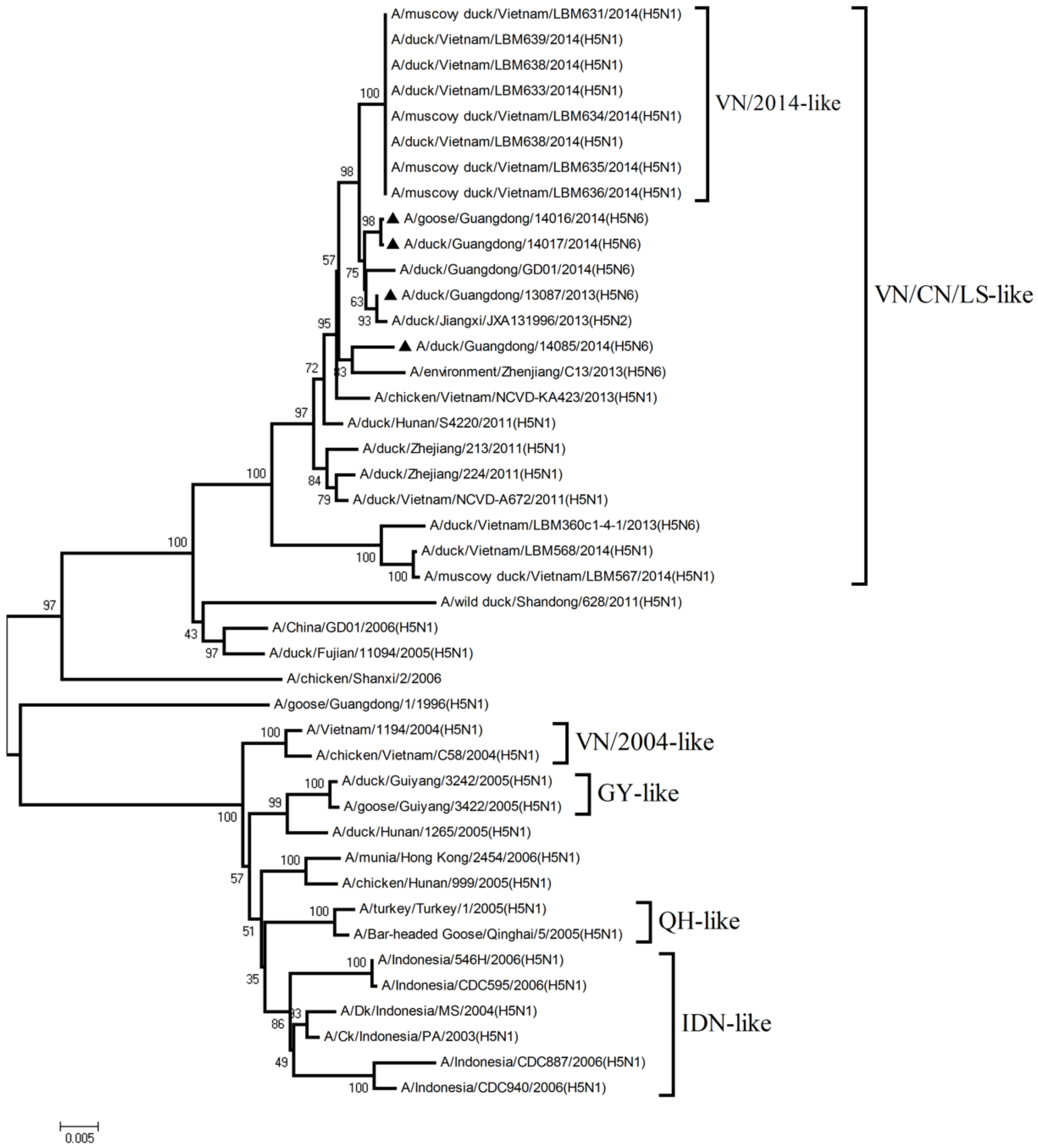
A/muscovy duck/Vietnam/LBM631/2014(H5N1)
A/duck/Vietnam/LBM639/2014(H5N1)
A/duck/Vietnam/LBM638/2014(H5N1)
A/duck/Vietnam/LBM633/2014(H5N1)
A/muscovy duck/Vietnam/LBM634/2014(H5N1)
A/duck/Vietnam/LBM638/2014(H5N1)
A/muscovy duck/Vietnam/LBM635/2014(H5N1)
A/muscovy duck/Vietnam/LBM636/2014(H5N1)
VN/2014-like
A/goose/Guangdong/14016/2014(H5N6)
A/duck/Guangdong/14017/2014(H5N6)
A/duck/Guangdong/GD01/2014(H5N6)
A/duck/Guangdong/13087/2013(H5N6)
A/duck/Jiangxi/JXA131996/2013(H5N2)
A/duck/Guangdong/14085/2014(H5N6)
A/environment/Zhenjiang/C13/2013(H5N6)
A/chicken/Vietnam/NCVD-KA423/2013(H5N1)
A/duck/Hunan/S4220/2011(H5N1)
A/duck/Zhejiang/213/2011(H5N1)
A/duck/Zhejiang/224/2011(H5N1)
A/duck/Vietnam/NCVD-A672/2011(H5N1)
A/duck/Vietnam/LBM360c1-4-1/2013(H5N6)
A/duck/Vietnam/LBM568/2014(H5N1)
A/muscovy duck/Vietnam/LBM567/2014(H5N1)
VN/CN/LS-like
A/wild duck/Shandong/628/2011(H5N1)
A/China/GD01/2006(H5N1)
A/duck/Fujian/11094/2005(H5N1)
A/chicken/Shanxi/2/2006
A/goose/Guangdong/1/1996(H5N1)
A/Vietnam/1194/2004(H5N1)
A/chicken/Vietnam/C58/2004(H5N1)
VN/2004-like
A/duck/Guiyang/3242/2005(H5N1)
A/goose/Guiyang/3422/2005(H5N1)
GY-like
A/duck/Hunan/1265/2005(H5N1)
A/munia/Hong Kong/2454/2006(H5N1)
A/chicken/Hunan/999/2005(H5N1)
A/turkey/Turkey/1/2005(H5N1)
A/Bar-headed Goose/Qinghai/5/2005(H5N1)
QH-like
A/Indonesia/546H/2006(H5N1)
A/Indonesia/CDC595/2006(H5N1)
A/Dk/Indonesia/MS/2004(H5N1)
A/Ck/Indonesia/PA/2003(H5N1)
A/Indonesia/CDC887/2006(H5N1)
A/Indonesia/CDC940/2006(H5N1)
IDN-like
0.005

Fig. S1D.

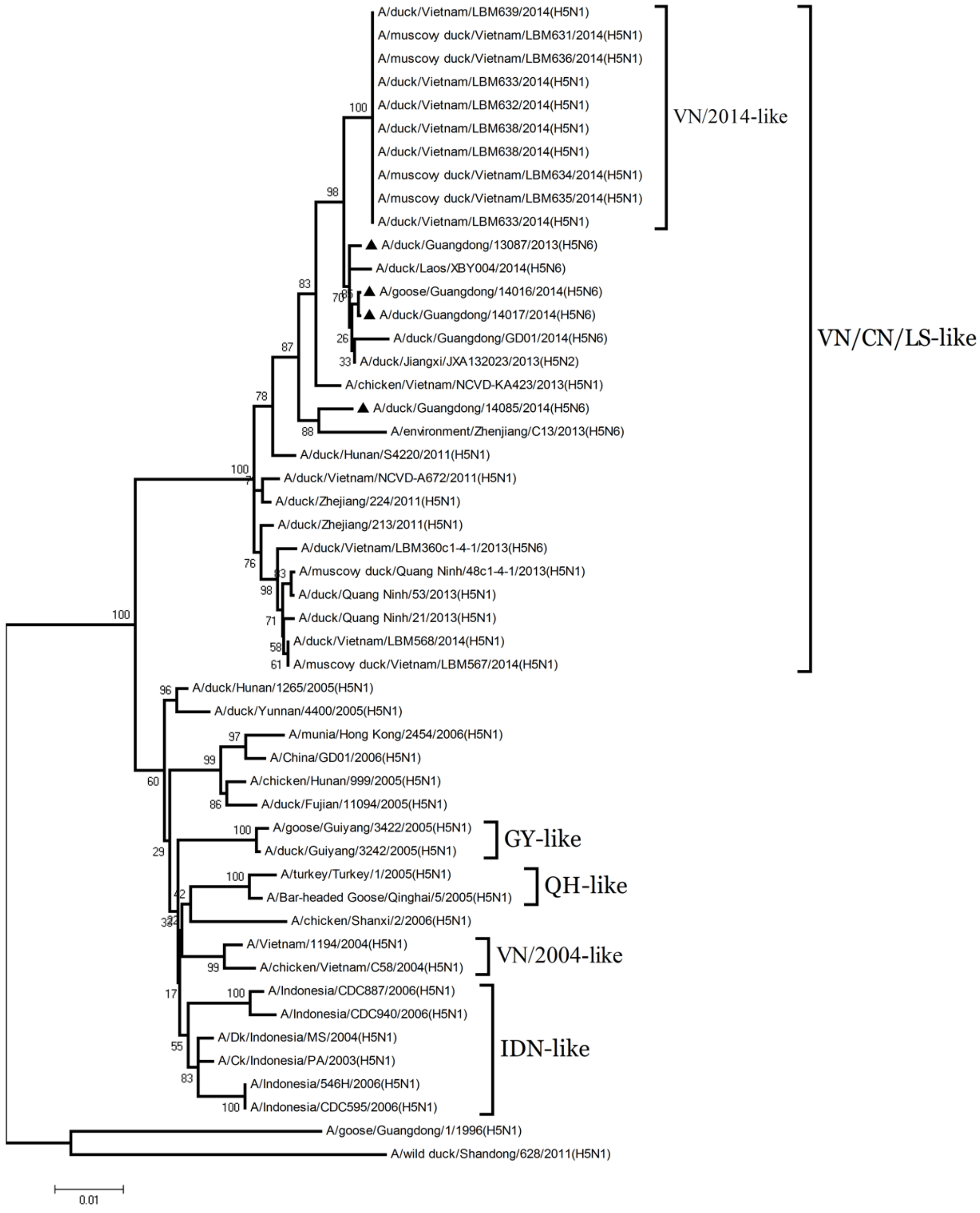

A/duck/Vietnam/LBM639/2014(H5N1)
A/muscovy duck/Vietnam/LBM631/2014(H5N1)
A/muscovy duck/Vietnam/LBM636/2014(H5N1)
A/duck/Vietnam/LBM633/2014(H5N1)
A/duck/Vietnam/LBM632/2014(H5N1)
A/duck/Vietnam/LBM638/2014(H5N1)
A/duck/Vietnam/LBM638/2014(H5N1)
A/muscovy duck/Vietnam/LBM634/2014(H5N1)
A/muscovy duck/Vietnam/LBM635/2014(H5N1)
A/duck/Vietnam/LBM633/2014(H5N1)
VN/2014-like
▲ A/duck/Guangdong/13087/2013(H5N6)
A/duck/Laos/XBY004/2014(H5N6)
▲ A/goose/Guangdong/14016/2014(H5N6)
▲ A/duck/Guangdong/14017/2014(H5N6)
A/duck/Guangdong/GD01/2014(H5N6)
A/duck/Jiangxi/JXA132023/2013(H5N2)
A/chicken/Vietnam/NCVD-KA423/2013(H5N1)
▲ A/duck/Guangdong/14085/2014(H5N6)
A/environment/Zhenjiang/C13/2013(H5N6)
A/duck/Hunan/S4220/2011(H5N1)
A/duck/Vietnam/NCVD-A672/2011(H5N1)
A/duck/Zhejiang/224/2011(H5N1)
A/duck/Zhejiang/213/2011(H5N1)
A/duck/Vietnam/LBM360c1-4-1/2013(H5N6)
A/muscovy duck/Quang Ninh/48c1-4-1/2013(H5N1)
A/duck/Quang Ninh/53/2013(H5N1)
A/duck/Quang Ninh/21/2013(H5N1)
A/duck/Vietnam/LBM568/2014(H5N1)
A/muscovy duck/Vietnam/LBM567/2014(H5N1)
VN/CN/LS-like
A/duck/Hunan/1265/2005(H5N1)
A/duck/Yunnan/4400/2005(H5N1)
A/munia/Hong Kong/2454/2006(H5N1)
A/China/GD01/2006(H5N1)
A/chicken/Hunan/999/2005(H5N1)
A/duck/Fujian/11094/2005(H5N1)
A/goose/Guiyang/3422/2005(H5N1)
A/duck/Guiyang/3242/2005(H5N1)
GY-like
A/turkey/Turkey/1/2005(H5N1)
A/Bar-headed Goose/Qinghai/5/2005(H5N1)
QH-like
A/chicken/Shanxi/2/2006(H5N1)
A/Vietnam/1194/2004(H5N1)
A/chicken/Vietnam/C58/2004(H5N1)
VN/2004-like
A/Indonesia/CDC887/2006(H5N1)
A/Indonesia/CDC940/2006(H5N1)
A/Dk/Indonesia/MS/2004(H5N1)
A/Ck/Indonesia/PA/2003(H5N1)
A/Indonesia/546H/2006(H5N1)
A/Indonesia/CDC595/2006(H5N1)
IDN-like
A/goose/Guangdong/1/1996(H5N1)
A/wild duck/Shandong/628/2011(H5N1)
0.01

Fig. S1E.

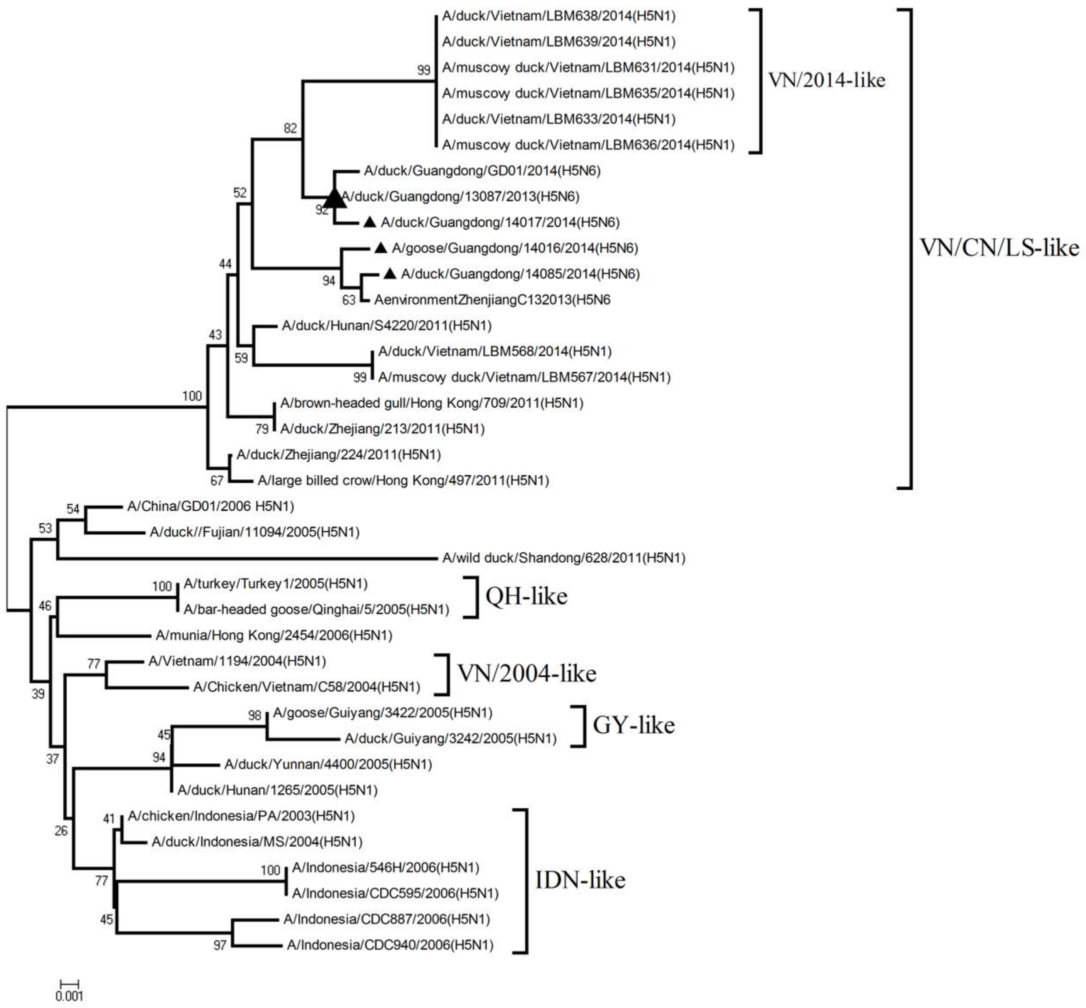
A/duck/Vietnam/LBM638/2014(H5N1)
A/duck/Vietnam/LBM639/2014(H5N1)
A/muscovy duck/Vietnam/LBM631/2014(H5N1)
A/muscovy duck/Vietnam/LBM635/2014(H5N1)
A/duck/Vietnam/LBM633/2014(H5N1)
A/muscovy duck/Vietnam/LBM636/2014(H5N1)
A/duck/Guangdong/GD01/2014(H5N6)
A/duck/Guangdong/13087/2013(H5N6)
A/duck/Guangdong/14017/2014(H5N6)
A/goose/Guangdong/14016/2014(H5N6)
A/duck/Guangdong/14085/2014(H5N6)
AenvironmentZhenjiangC132013(H5N6)
A/duck/Hunan/S4220/2011(H5N1)
A/duck/Vietnam/LBM568/2014(H5N1)
A/muscovy duck/Vietnam/LBM567/2014(H5N1)
A/brown-headed gull/Hong Kong/709/2011(H5N1)
A/duck/Zhejiang/213/2011(H5N1)
A/duck/Zhejiang/224/2011(H5N1)
A/large billed crow/Hong Kong/497/2011(H5N1)
A/China/GD01/2006 H5N1)
A/duck//Fujian/11094/2005(H5N1)
A/wild duck/Shandong/628/2011(H5N1)
A/turkey/Turkey1/2005(H5N1)
A/bar-headed goose/Qinghai/5/2005(H5N1)
A/munia/Hong Kong/2454/2006(H5N1)
A/Vietnam/1194/2004(H5N1)
A/Chicken/Vietnam/C58/2004(H5N1)
A/goose/Guiyang/3422/2005(H5N1)
A/duck/Guiyang/3242/2005(H5N1)
A/duck/Yunnan/4400/2005(H5N1)
A/duck/Hunan/1265/2005(H5N1)
A/chicken/Indonesia/PA/2003(H5N1)
A/duck/Indonesia/MS/2004(H5N1)
A/Indonesia/546H/2006(H5N1)
A/Indonesia/CDC595/2006(H5N1)
A/Indonesia/CDC887/2006(H5N1)
A/Indonesia/CDC940/2006(H5N1)
VN/2014-like
VN/CN/LS-like
QH-like
VN/2004-like
GY-like
IDN-like
99
82
52
92
44
94
63
43
59
99
100
79
67
54
53
100
46
77
39
98
45
94
37
41
26
100
77
45
97
0.001

Fig. S1F.

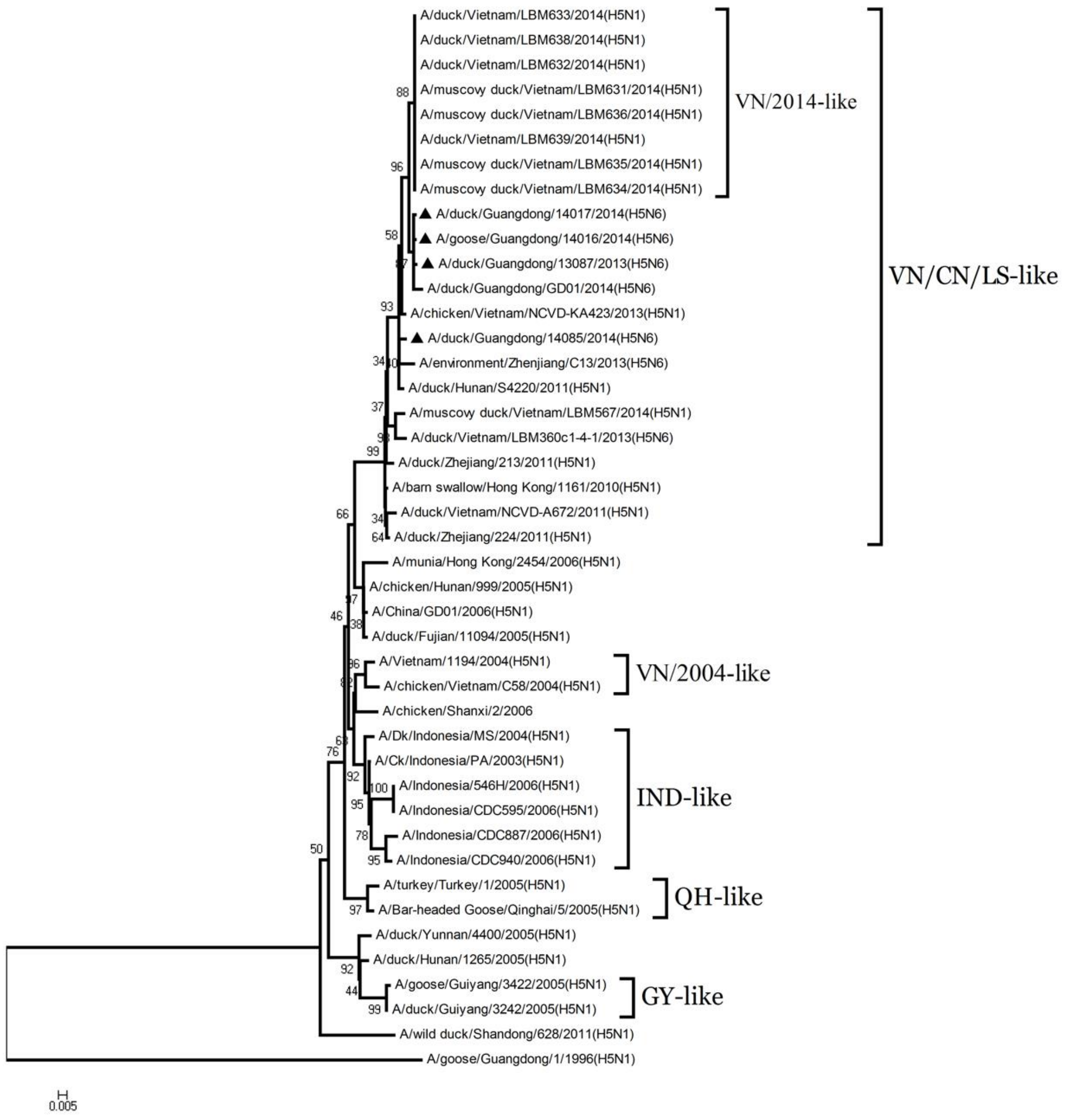
A/duck/Vietnam/LBM633/2014(H5N1)
A/duck/Vietnam/LBM638/2014(H5N1)
A/duck/Vietnam/LBM632/2014(H5N1)
A/muscovy duck/Vietnam/LBM631/2014(H5N1)
A/muscovy duck/Vietnam/LBM636/2014(H5N1)
A/duck/Vietnam/LBM639/2014(H5N1)
A/muscovy duck/Vietnam/LBM635/2014(H5N1)
A/muscovy duck/Vietnam/LBM634/2014(H5N1)
VN/2014-like
A/duck/Guangdong/14017/2014(H5N6)
A/goose/Guangdong/14016/2014(H5N6)
A/duck/Guangdong/13087/2013(H5N6)
A/duck/Guangdong/GD01/2014(H5N6)
A/chicken/Vietnam/NCVD-KA423/2013(H5N1)
A/duck/Guangdong/14085/2014(H5N6)
A/environment/Zhenjiang/C13/2013(H5N6)
A/duck/Hunan/S4220/2011(H5N1)
A/muscovy duck/Vietnam/LBM567/2014(H5N1)
A/duck/Vietnam/LBM360c1-4-1/2013(H5N6)
A/duck/Zhejiang/213/2011(H5N1)
A/barn swallow/Hong Kong/1161/2010(H5N1)
A/duck/Vietnam/NCVD-A672/2011(H5N1)
A/duck/Zhejiang/224/2011(H5N1)
VN/CN/LS-like
A/munia/Hong Kong/2454/2006(H5N1)
A/chicken/Hunan/999/2005(H5N1)
A/China/GD01/2006(H5N1)
A/duck/Fujian/11094/2005(H5N1)
A/Vietnam/1194/2004(H5N1)
A/chicken/Vietnam/C58/2004(H5N1)
VN/2004-like
A/chicken/Shanxi/2/2006
A/Dk/Indonesia/MS/2004(H5N1)
A/Ck/Indonesia/PA/2003(H5N1)
A/Indonesia/546H/2006(H5N1)
A/Indonesia/CDC595/2006(H5N1)
A/Indonesia/CDC887/2006(H5N1)
A/Indonesia/CDC940/2006(H5N1)
IND-like
A/turkey/Turkey/1/2005(H5N1)
A/Bar-headed Goose/Qinghai/5/2005(H5N1)
QH-like
A/duck/Yunnan/4400/2005(H5N1)
A/duck/Hunan/1265/2005(H5N1)
A/goose/Guiyang/3422/2005(H5N1)
A/duck/Guiyang/3242/2005(H5N1)
GY-like
A/wild duck/Shandong/628/2011(H5N1)
A/goose/Guangdong/1/1996(H5N1)
88
96
58
93
34
37
99
66
34
64
97
46
38
96
82
63
76
92
100
95
78
95
50
97
92
44
99
0.005
